# Bacterial Neuraminidase Rescues Influenza Virus Replication from Inhibition by a Neuraminidase Inhibitor

**DOI:** 10.1371/journal.pone.0045371

**Published:** 2012-09-18

**Authors:** Tomoko Nishikawa, Kazufumi Shimizu, Torahiko Tanaka, Kazumichi Kuroda, Tadatoshi Takayama, Tatsuo Yamamoto, Nobuhiro Hanada, Yoshiki Hamada

**Affiliations:** 1 SRBD Project, Nihon University School of Medicine, Itabashi-ku, Tokyo, Japan; 2 Division and Department of Obstetrics and Gynecology, Nihon University School of Medicine, Itabashi-ku, Tokyo, Japan; 3 Division of Biochemistry, Department of Biomedical Sciences, Nihon University School of Medicine, Itabashi-ku, Tokyo, Japan; 4 Division of Microbiology, Department of Pathology and Microbiology, Nihon University School of Medicine, Itabashi-ku, Tokyo, Japan; 5 Division of Digestive Surgery, Department of Surgery, Nihon University School of Medicine, Itabashi-ku, Tokyo, Japan; 6 Department of Translational Research, Tsurumi University School of Dental Medicine, Turumi-ku, Yokohama, Japan; 7 Department of Oral & Maxillofacial Surgery, Tsurumi University School of Dental Medicine, Turumi-ku, Yokohama, Japan; German Primate Center, Germany

## Abstract

Influenza virus neuraminidase (NA) cleaves terminal sialic acid residues on oligosaccharide chains that are receptors for virus binding, thus playing an important role in the release of virions from infected cells to promote the spread of cell-to-cell infection. In addition, NA plays a role at the initial stage of viral infection in the respiratory tract by degrading hemagglutination inhibitors in body fluid which competitively inhibit receptor binding of the virus. Current first line anti-influenza drugs are viral NA-specific inhibitors, which do not inhibit bacterial neuraminidases. Since neuraminidase producing bacteria have been isolated from oral and upper respiratory commensal bacterial flora, we posited that bacterial neuraminidases could decrease the antiviral effectiveness of NA inhibitor drugs in respiratory organs when viral NA is inhibited. Using *in vitro* models of infection, we aimed to clarify the effects of bacterial neuraminidases on influenza virus infection in the presence of the NA inhibitor drug zanamivir. We found that zanamivir reduced progeny virus yield to less than 2% of that in its absence, however the yield was restored almost entirely by the exogenous addition of bacterial neuraminidase from *Streptococcus pneumoniae*. Furthermore, cell-to-cell infection was severely inhibited by zanamivir but restored by the addition of bacterial neuraminidase. Next we examined the effects of bacterial neuraminidase on hemagglutination inhibition and infectivity neutralization activities of human saliva in the presence of zanamivir. We found that the drug enhanced both inhibitory activities of saliva, while the addition of bacterial neuraminidase diminished this enhancement. Altogether, our results showed that bacterial neuraminidases functioned as the predominant NA when viral NA was inhibited to promote the spread of infection and to inactivate the neutralization activity of saliva. We propose that neuraminidase from bacterial flora in patients may reduce the efficacy of NA inhibitor drugs during influenza virus infection. (295 words).

## Introduction

Influenza is one of the most common infectious diseases, affecting millions of people around the world every year. Occasionally, it causes a catastrophic pandemic such as the “Spanish flu” in 1918, which killed 30–50 million people worldwide. The most effective means of protection against influenza is vaccination; however, its effectiveness has been limited because etiological influenza A and B viruses constantly undergo antigenetic change. Moreover, the time needed to prepare a vaccine against a newly isolated influenza virus is more than half a year. This makes an emergency vaccine preparation against a pandemic influenza virus, such as the 2009 pandemic, difficult. However, as a vaccine alternative, several anti-influenza drugs have been developed. The drugs are categorized into two groups, M2 protein inhibitors and neuraminidase inhibitors. The former was developed earlier and most influenza viruses presently circulating among humans are resistant against the inhibitors from this group. In the latter, oseltamivir [1] and zanamivir [2] are widely used against influenza, effectively reducing the duration and severity of influenza illness. These drugs were the only available options during the 2009 pandemic.

Influenza type A and B viruses contain three major surface proteins, HA (hemagglutinin), NA (neuraminidase) and M2 (membrane protein 2). HA mediates viral attachment to host cells by binding sialic acids on carbohydrate side chains of cell surface glycoproteins and glycolipids. HA also mediates virus entry into host cells through the fusion of the viral envelope with the endosomal membrane [3]. As fusion occurs, the viral genome is released into cytoplasm of host cells by the aid of the M2 protein ion channel. NA cleaves the terminal sialic acid residues on oligosaccharide chains that serve as viral HA receptors. Through this enzymatic activity, NA plays an important role in the spread of infection from cell to cell because virions stick to the cell surface or aggregate with each other if sialic acid residues are not removed from the surface of infected cells and progeny viruses [4]. In body fluids, numerous molecules containing sialic acid exist and most of them are able to bind to virus HA and inhibit the hemagglutination activity of influenza virus. Human saliva has been reported to contain such hemagglutination inhibitors [5]–[7]. During the initial infection of mucosal epithelial cells, influenza virus encounters these inhibitory molecules in mucus and viral NA is speculated to inactivate such inhibitors so that viral HA is able to bind to receptors on the surface of epithelial cells [8].

Influenza virus initiates infection in the upper respiratory tract where commensal bacterial flora exists. The synergism between influenza virus and bacteria has been documented in past influenza outbreaks. It was first observed when the swine influenza virus was discovered by Shope in 1931. He indicated that the isolated virus and *Haemophilus influenzae* acted together to produce swine influenza and that neither alone was capable of inducing disease [9]. Furthermore, reexamination of samples from the influenza pandemic of 1918 indicated that the majority of patients died of secondary bacterial pneumonia [10]–[12]. In the influenza pandemic of 1957–1958, most deaths attributed to influenza A virus infection occurred concurrently with bacterial pneumonia [13]. Moreover, recent postmortem studies among fatal A(H1N1)pdm09 cases from the 2009 pandemic established a link between bacterial lung infections and increased mortality [14] or developing complications [15].

Mechanisms for the synergy between bacteria and influenza viruses involve the activity of either bacterial or viral enzymes. For influenza virus to obtain membrane fusion activity, HA protein has to be cleaved by a host proteinase. Some strains of *Staphylococcus aureus* secrete a protease which significantly influences the outcome of influenza infection by cleavage activation of HA [16], [17]. Influenza virus NA, on the other hand, potentiates the development of pneumonia by stripping sialic acid from lung cells, thus exposing receptors for *Streptococcus pneumoniae* adhesion [18], [19].

Classical studies on influenza virus receptors by Gottschalk showed that neuraminidase treatment inactivates hemagglutination inhibitors in serum and mucus secretions by removing the sialic acid residues of oligosaccharide chains on the inhibitors [20]. The most well-known source of neuraminidase used for this purpose is a so-called receptor-destroying enzyme (RDE, crude filtrates of *Vibrio cholerae* culture fluid) [21]. It has been shown by several groups that influenza A viruses lacking neuraminidase activity can undergo multiple cycles of replication in an *in vitro* infection system if bacterial neuraminidase is provided exogenously [22], [23]. In this manner, viral NA becomes dispensable because bacterial neuraminidase assumes its role and makes up for its absence to promote virus infection. Several species of bacteria isolated from oral and respiratory tract bacterial flora have been reported to secrete proteins possessing neuraminidase activity [24]–[30].

Since anti-influenza drugs targeting NA are specific to influenza virus NA, they do not inhibit bacterial neuraminidases at the concentration prescribed to patients. We posited that neuraminidase derived from bacterial flora found in patients could compensate for inhibited viral NA and decrease the antiviral effectiveness of these drugs. In the present study, we examined the effects of bacterial neuraminidase on influenza virus infection in the presence of an NA inhibitor (zanamivir) in an *in vitro* model of infection. Our data implicate bacterial neuraminidase in the reduction of antiviral efficacy of this class of drugs.

## Results

### Screening of Neuraminidase-secreting Oral and Upper Respiratory Tract Bacteria

**Figure 1:**
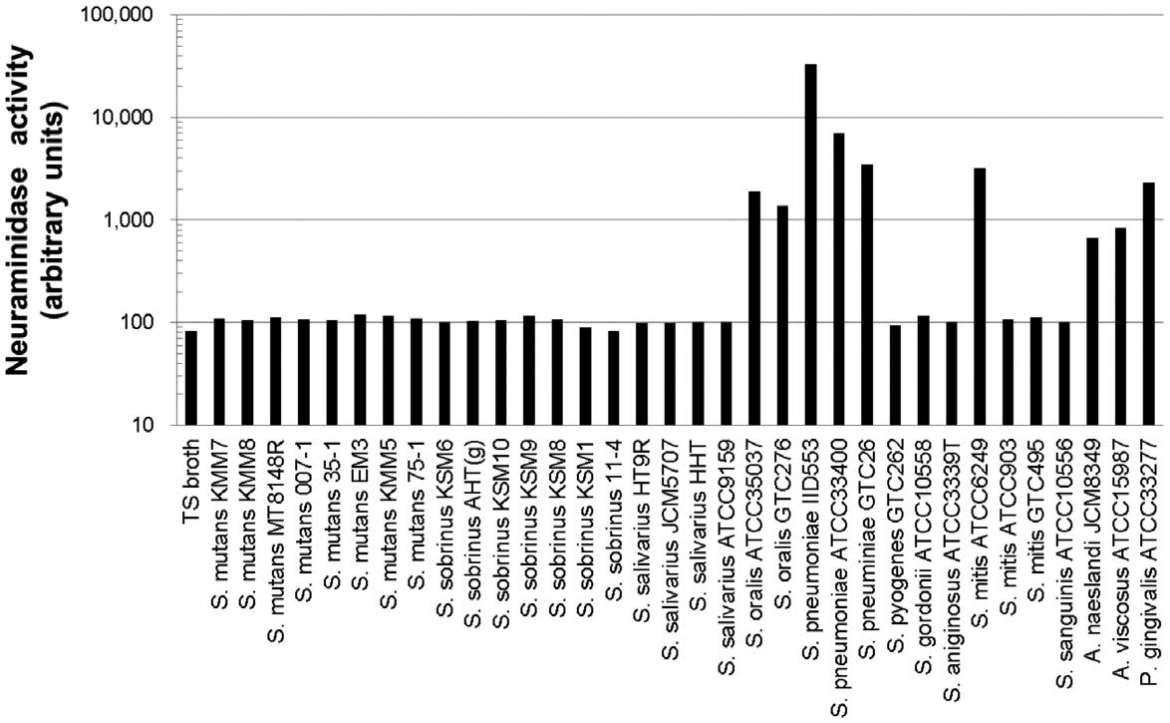
Screening of neuraminidase-secreting oral and upper respiratory bacteria. Neuraminidase activity of bacterial culture supernatants was measured and expressed as arbitrary units of luminescence signals. TS broth was the culture media used for all bacteria cultures in this study. The value for TS broth alone was assumed to be background noise.

The bacterial culture supernatants of 34 strains of 13 species isolated from human oral or upper respiratory tracts were screened for secreted neuraminidase activity (**Figure 1**). Nine strains of 6 species; *Streptococcus oralis*, *Streptococcus pneumoniae*, *Streptococcus mitis, Actinomyces naeslundii*, *Actinomyces viscosus, and Porphyromonas gingivalis* were positive for the activity. Among them *S. pneumoniae* (IID553) exhibited the highest activity and, therefore, the culture supernatant was used in subsequent experiments. On the other hand, *Streptococcus mutans* (8 strains), *Streptococcus sobrinus* (7 strains), *Streptococcus salivarius* (4 strains), *Streptococcus pyogenes* (1 strain), *Streptococcus gordonii* (1 strain), *Streptococcus anginosus* (1 strain), and *Streptococcus sanguinis* (1 strain) were negative for secreted neuraminidase activity.

To evaluate the level of neuraminidase activity of *S. pneumoniae*, we compared it with a highly purified neuraminidase from *Arthrobacter ureafaciens,* which has a known activity, and designated it as the standard neuraminidase (**Table 1**). The neuraminidase activity of *S. pneumoniae* culture supernatant was calculated to be130 µunits/ml compared with the standard. We further measured the neuraminidase activity of influenza A/Udorn/72 virus suspension (320 HAU/ml, this is the usual level of virus concentration in culture medium of infected MDCK cells), influenza B/Johannesburg/99 (160 HAU/ml), human saliva samples, and *Vibrio cholerae* RDE (receptor destroying enzyme, the most well-known source of neuraminidase) (**Table 1**). The neuraminidase activity of *S. pneumoniae* was sufficient, exhibiting 30% of A/Udorn/72 activity. Saliva also possessed neuraminidase activity which was 11% of the virus activity. B/Johannesburg/99 virus suspension and RDE showed about 32-fold and 8.5-fold higher activity than that of A/Udorn/72, respectively.

**Table 1:** Comparison of neuraminidase activities with those of A/Udorn/72 virus.

Sample	Neuraminidase activity		Calculated original activity	
	Dilution	Signals^a^ ± SD^b^	µunit/ml^c^ ± SD^b^	Ratio to A/Udorn
*Arthrobacter ureafaciens* neuraminidase (1 unit/ml)	×10,000	69,258±1,262	1,000,000	2,300
*Vibrio cholerae* culture supernatant (RDE)	×100	25,592±9,516	3,700±1,400	8.5
*Streptococcus pneumonia* culture supernatant	×1	89,954±4,574	130±6.6	0.30
Human saliva	×1	33,283±1,994	48±2.9	0.11
B/Johannesburg/99 virus (160 HAU/ml)	×250	39,026±1,230	14,000±440	32
A/Udorn/72(H3N2) virus (320 HAU/ml)	×10	30,303±926	440±13	1.00

a mean arbitrary units of luminescence signals.

b SD, standard deviation.

c calculated using a standard curve obtained by purified *Arthrobacter ureafaciens* neuraminidase of known unit activity.

### Zanamivir Specifically Inhibits Influenza Virus Neuraminidase

**Figure 2:**
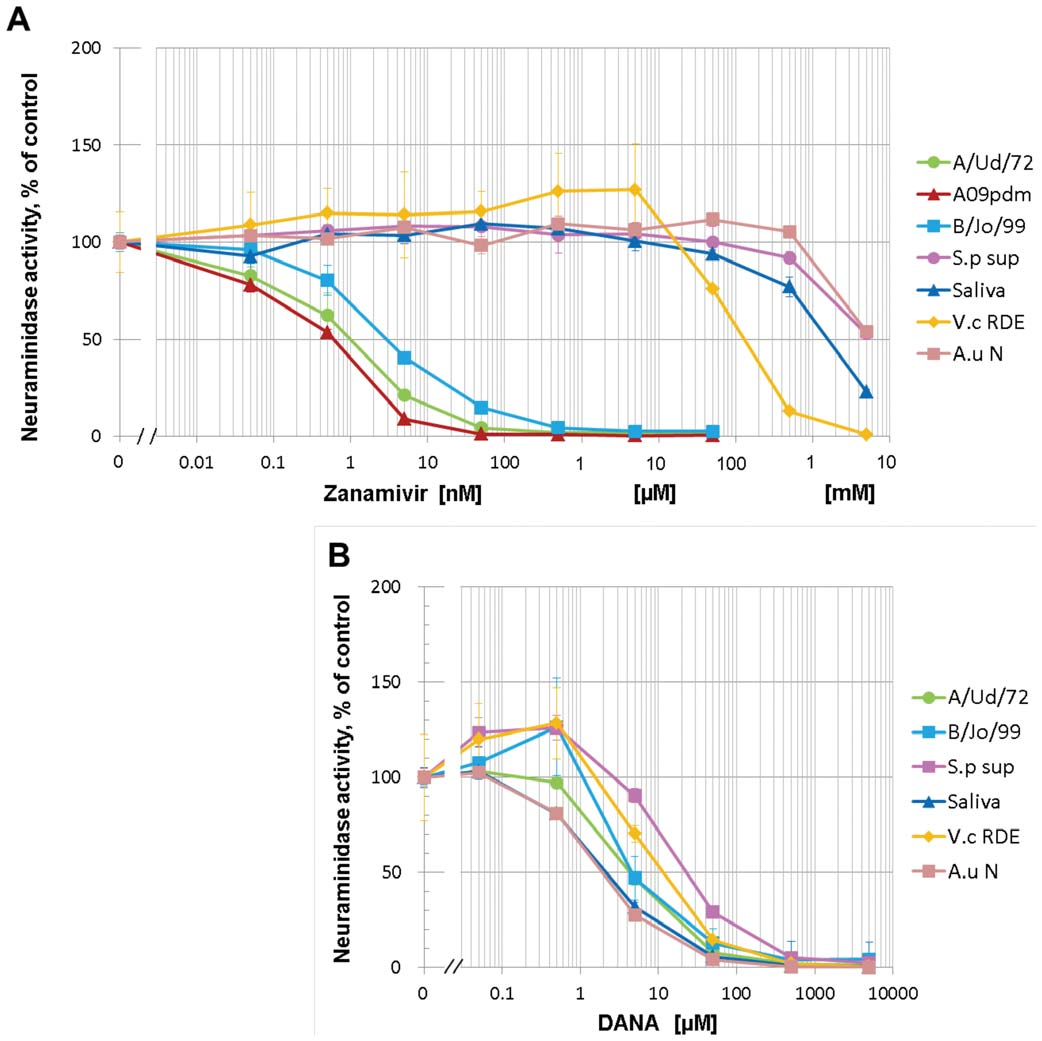
Sensitivity of neuraminidases from influenza viruses, bacteria and saliva against zanamivir and DANA. Neuraminidase activity of virus, bacteria and saliva was assayed in the presence of ten-fold serial dilutions of zanamivir (an anti-influenza NA drug) (**A**) or DANA (2-Deoxy-2,3-dehydro-N-acetylneuraminic acid; a neuraminidase inhibitor reagent) (**B**). Neuraminidase activity was expressed as percentage of control activity without zanamivir and DANA. Values were the mean and standard deviation of triplicate measurements. Zanamivir inhibited virus neuraminidases with an IC_50_ of 0.6–3 nM and bacteria and saliva neuraminidases with an IC_50_ of 0.1–5 mM. DANA inhibited neuraminidases with an IC_50_ of 2–20 µM irrespective of the source.

We employed zanamivir as a representative of the anti-influenza NA inhibitors and measured the dose-dependent inhibition by zanamivir on neuraminidase activity of influenza viruses (A/Udorn/72(H3N2), A/Chiba/2009(H1N1)pdm and B/Johannesburg/99), *S. pneumoniae* and saliva, and on standard bacterial neuraminidases from *A. ureafaciens* and *V. cholerae* RDE (**Figure 2A**). Zanamivir inhibited neuraminidase activity of influenza viruses with 0.5–3 nM IC_50_ values, whereas bacterial and salivary neuraminidases were inhibited by the drug with IC_50_ values ranging from 0.1–5 mM. Thus, zanamivir selectively inhibited influenza virus NA with approximately a million-fold higher potency than that against bacterial neuraminidases. In contrast to zanamivir, DANA (2-deoxy-2,3-dehydro-N-acetylneuraminic acid), one of oldest known synthetic sialic acid analogues, similarly inhibited viral and bacterial neuraminidase activity. The IC_50_ values by DANA ranged from about 2 to 20 µM among the tested neuraminidases (**Figure 2B**), indicating that DANA inhibited the viral and bacterial neuraminidases equally. Based on these dose-dependent inhibition results, 250 nM zanamivir was used for specific inhibition of viral neuraminidases and 2.5 mM DANA was used for nonspecific inhibition of viral and bacterial neuraminidases in the following experiments.

### Effects of Bacterial Neuraminidases on the Suppression of Virus Growth by Zanamivir

**Figure 3:**
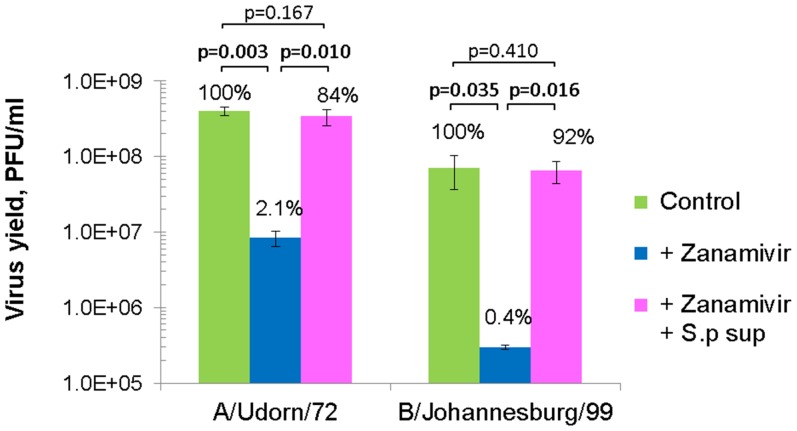
Bacterial neuraminidase restores the growth of influenza virus from suppression by zanamivir. A/Udorn/72 and B/Johannesburg/99 viruses were inoculated onto MDCK cells at a MOI of 0.001 and incubated with MEM containing 250 nM zanamivir in the presence or absence of *Streptococcus pneumoniae* culture supernatant (final 6 µunits/ml neuraminidase activity). Culture media were harvested at 40 hpi and the virus titers were determined by plaque assay. Data were obtained from triplicate samples from three wells and expressed as the mean with the standard deviation. Differences between groups were examined for statistical significance using Welch’s *t*-test. The p-value calculated using a one-tailed test was presented on the figure.

**Figure 4:**
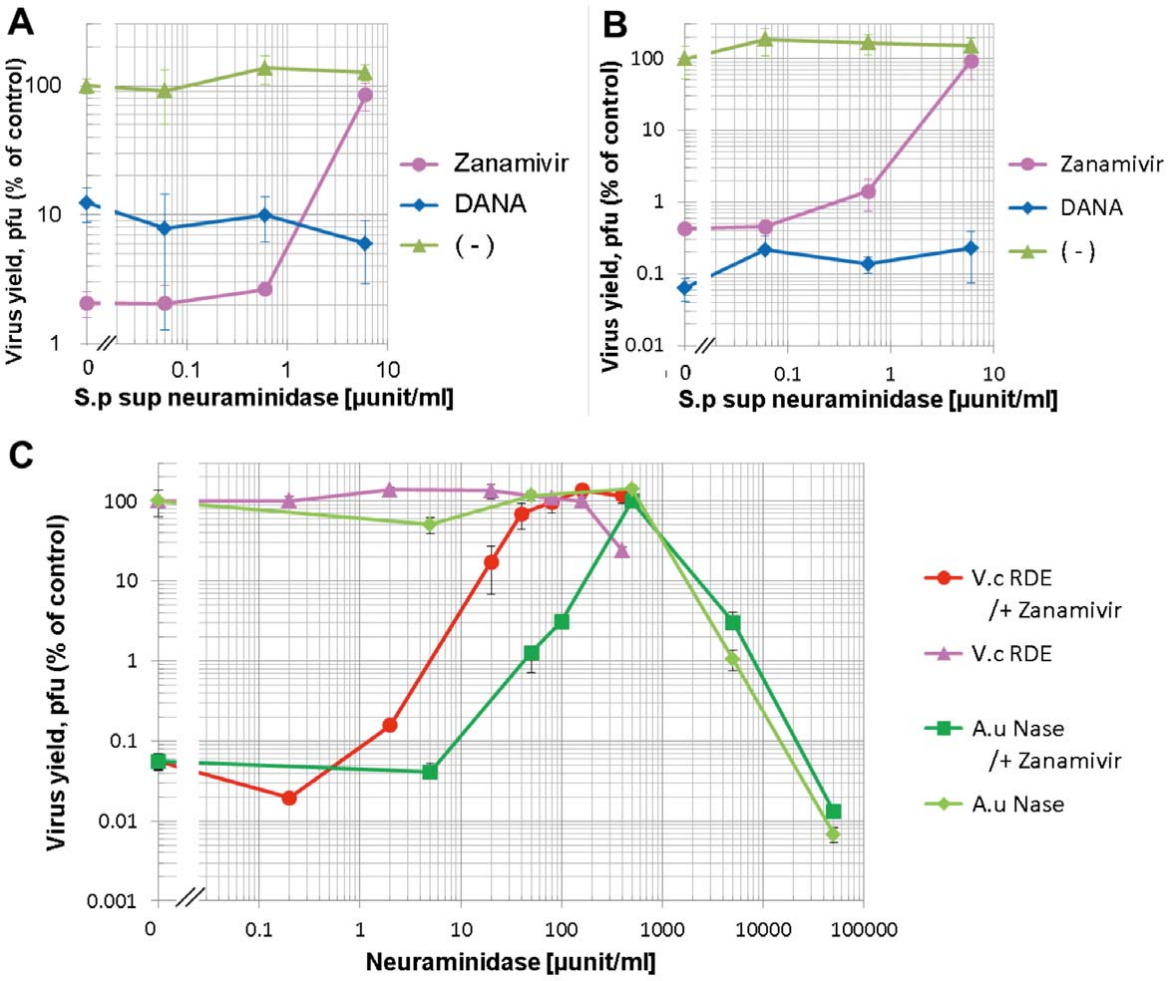
Dose dependent effects of bacterial neuraminidase on the growth of influenza virus in the presence of NA Inhibitors. A/Udorn/72 (**A** and **C**) and B/Johannesburg/99 (**B**) viruses were inoculated at a MOI of 0.001 and cells were incubated at 37°C in 5% CO_2_ in MEM containing various amounts (x-axis) of bacterial neuraminidase (*S.p* sup (**A** and **B**), *Streptococcus pneumoniae* culture supernatant; *V.c* RDE (**C**), *Vibrio cholerae* RDE; *A.u* Nase (**C**), purified neuraminidase from *Arthrobacter ureafaciens*) with 250 nM zanamivir (+ Zanamivir; **A**, **B**, and **C**) or 2.5 mM DANA (+ DANA, **A** and **B**). Culture media were harvested at 40 hpi and the virus titers were determined by plaque assay. Data were individually obtained from triplicate samples and expressed as means with standard deviations.

The highly specific inhibition by zanamivir against influenza virus neuraminidases enabled us to assess the effect of bacterial neuraminidase on influenza virus infection. A/Udorn/72 and B/Johannesburg/99 viruses were inoculated onto MDCK cells at a multiplicity of infection (MOI) of 0.001 and incubated with MEM containing 250 nM zanamivir in the presence or absence of *Streptococcus pneumoniae* culture supernatant at a final concentration of 6 µunits/ml neuraminidase activity. Culture media were harvested at 40 hpi and the virus titers were determined by plaque assay. Zanamivir suppressed the yield of progeny virus from A/Udorn/72-infected cells to 2% of the control (**Figure 3**). Remarkably, the yield was restored to 84% by the inclusion of *S. pneumoniae* culture supernatant. Similarly, neuraminidase from *S. pneumoniae* restored the yield of B/Johannesburg/99 virus from the potent inhibition by zanamivir. These results clearly indicated that the bacterial neuraminidase compensated for the virus NA activity in the presence of an influenza NA inhibitor. To clarify this compensation effect in more detail, dose responses of the *S. pneumoniae* culture supernatant on influenza A/Udorn/72 and B/Johannesburg/99 virus yields were tested in the presence or absence of NA inhibitors (**Figures 4A and 4B**, respectively). Interestingly, *S. pneumoniae* culture supernatant slightly increased the virus production for both influenza A and B viruses in the absence of NA inhibitor. The inhibitory effect of zanamivir (250 nM) on virus production was diminished by increasing concentrations of *S. pneumoniae* culture supernatant. At 6 µunits/ml of *S. pneumoniae* neuraminidase activity, virus yields were completely restored for both A and B viruses. The nonspecific neuraminidase inhibitor DANA (2.5 mM) also inhibited influenza virus production but this inhibition was not restored by the addition of *S. pneumoniae* culture supernatant. This is most likely attributed to the dual inhibitory activity of DANA against both influenza virus and *S. pneumoniae* neuraminidases. We further confirmed the restoring effect of bacterial neuraminidase by using neuraminidases from *V. cholerae* (RDE) and *A. ureafaciens* (**Figure 4C**). Both bacterial neuraminidases diminished the inhibitory effect of zanamivir on A/Udorn/72 production. It is worth noting that high doses of exogenous neuraminidase (more than 500 µunits/ml) alone decreased virus yields. This inhibition may have been caused by the depletion of virus receptors on the host MDCK cells.

### Effects of Bacterial Neuraminidases on the Suppression of Virus Spread by Zanamivir

**Figure 5:**
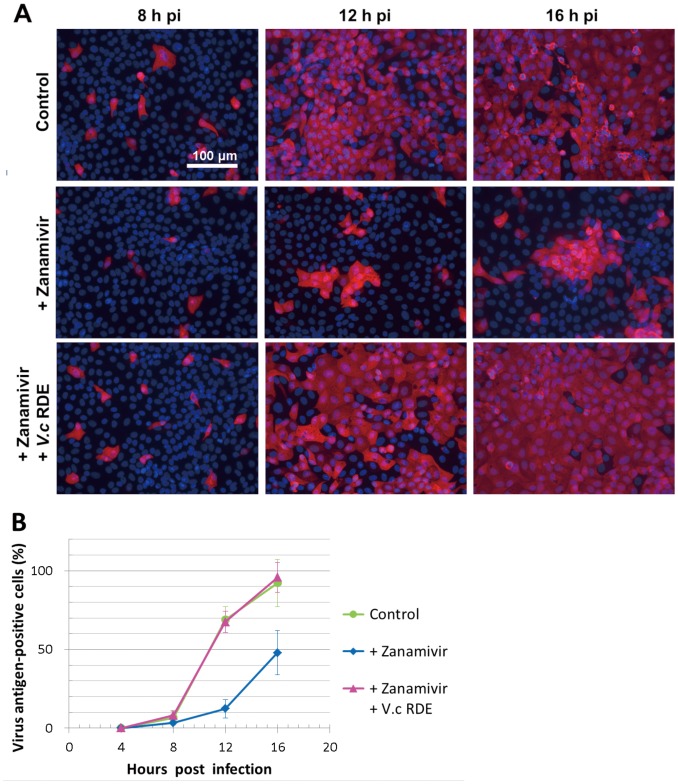
Bacterial neuraminidase restores the spread of infection from the inhibition by zanamivir. A/Udorn/72 virus was inoculated at a MOI of 0.01 onto MDCK cells and incubated for 4, 8, 12, and 16 h at 37°C in MEM containing 250 nM zanamivir (+ Zanamivir) with or without *Vibrio cholerae* RDE (+ V.c RDE, 20 µunits/ml neuraminidase activity). Virus antigens were stained by indirect immunofluorescence using rabbit polyclonal antibody against purified A/Udorn/72 virions. Nuclei of all cells were counterstained by Hoechst 33342. The stained cells were observed using a fluorescence microscope (BZ-8000, Keyence, Osaka, Japan). Depicted (**A**) is the merged image of virus antigen staining (red) and nucleus DNA counterstaining (blue). All panels are at the same magnification, and the scale bar indicates 100 µm. Virus antigen-positive or –negative cells and total cells of nucleus DNA staining were separately counted using the BZ-8000 attached software and the ratio (%) of virus antigen-positive cells are plotted according to incubation times(**B**).

The cell-to-cell spread of infection and its suppression by zanamivir was evaluated by immunofluorescence analysis. A/Udorn/72 virus was inoculated at a MOI of 0.01 onto MDCK cells grown on coverslips, and cells were incubated for 4, 8, 12, and 16 h at 37°C in MEM containing 250 nM zanamivir with or without *V. cholerae* RDE (20 µunits/ml neuraminidase activity) and then stained with anti-A/Udorn/72 antibody. In control cells (in the absence of both zanamivir and bacterial neuraminidase), antigen-positive cells increased according to incubation times and the majority (70%) of the cells became positive at 12 hpi, indicating cell-to-cell spread of virus infection (**Figure 5**, top). In the presence of zanamivir, only small portion of cells (12%, **Figure 5B**) became antigen-positive at 12 hpi. In contrast, in the presence or absence of zanamivir, the number of positive cells at 4 hpi was the same. These results clearly suggest that the spread of infection was severely suppressed by zanamivir but the initial infection was not (**Figure 5**, middle). However, when *V. cholerae* RDE was present in addition to zanamivir, the majority of cells (68%, **Figure 5B**) were antigen-positive at 12 hpi, indicating that the presence of RDE diminished the inhibitory effect of zanamivir and restored the cell-to-cell spread of infection (**Figure 5**, bottom).

### Inactivation of Hemagglutination Inhibition Activity of Saliva by Neuraminidase Treatment

Hemagglutination activity of viruses reflects their receptor-binding activity. We detected significant inhibitory activity in human saliva against hemagglutination by influenza viruses. Saliva samples from three healthy donors were tested for hemagglutination inhibition (HI) activity against three strains of A (H3N2) virus, three strains of A (H1N1) virus and two strains of B virus (**Table 2**). HI titers against A type viruses varied considerably among donors. H3N2 subtype viruses tended to be more resistant to saliva than H1N1 subtype viruses and saliva HI titers of Donor 3 were under the detection limit of two against H3N2 viruses. The saliva samples exhibited the highest HI titer of 4,096 against B/Johannesburg/99, which was the most sensitive to the inhibitory activity of saliva.

**Table 2:** Hemagglutination inhibition activity of human saliva against influenza virus.

Indicator virus	Hemagglutination inhibition titer					
	Donor 1		Donor 2		Donor 3	
	RDE treatment^a^		RDE treatment		RDE treatment	
	−	+	−	+	−	+
A/Udorn/307/72(H3N2)	**64**	**<4**	**32**	**<4**	**<2**	**<4**
A/Kitakyushu/93(H3N2)	**16**	**<4**	**96**	**<4**	**<2**	**<4**
A/Panama/2007/99(H3N2)	**4**	ND^b^	**4**	ND	**<2**	ND
A/Puerto Rico/8/34(H1N1)	**192**	**<4**	**192**	**<4**	**16**	**<4**
A/Yamagata/120/86(H1N1)	**8**	ND	**512**	ND	**<2**	ND
A/New Caledonea/20/99(H1N1)	**192**	**<4**	**2,048**	**<4**	**256**	**<4**
B/Johannesburg/5/99	**1,024**	**<4**	**4,096**	**<4**	**2,048**	**<4**
B/Kyoto/KU37/2011	**32**	**<4**	**<2**	ND	**<2**	ND

a RDE treatment, treated with an equal volume of *Vibrio cholerae* RDE at 37°C for 16 h, followed by heating at 56°C for 30 minutes to inactivate the enzyme.

b ND, not determined.

Next, we tested effect of bacterial neuraminidase on saliva HI activity (**Table 2**). Saliva samples were incubated with *V. cholerae* RDE at 37°C for 16 h, followed by heating at 56°C for 30 min to inactivate the enzyme, and the remaining HI titers were determined. As shown in **Table 2**, the HI activity of saliva was completely inactivated by RDE treatment. We confirmed that heating at 56°C for 30 min did not decrease the HI titer of saliva (data not shown), indicating that the HI ability of saliva was neuraminidase-sensitive and heat-stable. We also determined the HI titer of serum from the three saliva donors after standard RDE treatment (data not shown). In contrast to saliva HI activity, serum HI activity was resistant to RDE treatment and HI titers against B/Johannesburg/99 virus were 2 to 10 fold lower than that of corresponding saliva, confirming that saliva HI activity is not due to serum antibodies against influenza virus.

### Effect of Zanamivir on Hemagglutination Inhibition Activity of Saliva in the Presence or Absence of Bacterial Neuraminidase

Since the saliva HA inhibitor is inactivated by *V. cholerae* RDE neuraminidase, we assumed that viral NA was also capable of reducing the inhibitor activity. If so, then zanamivir antagonism of viral NA should result in a more potent HA inhibitor activity. To examine this possibility, the effect of zanamivir on HI activity of saliva was examined in the presence or absence of the bacterial neuraminidase RDE. Two-fold serial dilutions of a saliva sample from one healthy donor were mixed with equal volumes of virus suspension (8 HAU/ml) containing or not containing 500 nM zanamivir, and with or without RDE (148 µunits/ml neuraminidase activity). After one hour incubation at 37°C, chicken red blood cells were added and the samples were incubated at 4°C. Finally, one hour later the HI titer was determined.

**Figure 6:**
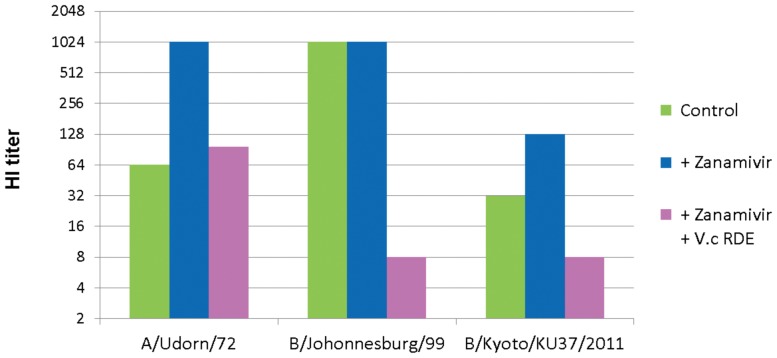
Bacterial neuraminidase diminishes the enhancement of hemagglutination inhibition activity of saliva by zanamivir. Two-fold serial dilutions (25 µl) of a saliva sample was mixed with an equal volume of A/Udorn/72, B/Johonnesburg/99 and B/Kyoto/KU37/2011 virus suspensions (8 HAU/ml) containing zanamivir (+ Zanamivir, 500 nM) with or without *Vibrio cholerae* RDE (+ *V.c* RDE, 150 µunits/ml neuraminidase activity) and incubated at 37°C for 60 min. Then 50 µl of 0.5% chicken red blood cells was added, incubated at 4°C for 60 min, and hemagglutination was read. HI titers are reciprocals of the highest dilution of samples that inhibited hemagglutination.

The tested saliva exhibited an HI titer of 64 against A/Udorn/72 virus in the absence of zanamivir (**Figure 6** **)**, whereas the presence of zanamivir induced a 16-fold increase in the HI titer to 1,024. This increase, however, was diminished by the presence of RDE. Based on these results, we reasoned that viral NA inactivates the HA inhibitor in saliva to some extent and bacterial neuraminidase is capable of supporting viral NA or even complementing its activity in the presence of zanamivir. Interestingly, the HI titer against B/Johannesburg/99 virus (1, 024) was not affected by zanamivir treatment, yet RDE decreased the HI titer to 8, a 128-fold decrease, suggesting that saliva HA inhibitors are resistant to B/Johannesburg/99 NA but sensitive to *V. cholerae* RDE. Saliva HI titer of another B type virus, B/Kyoto/KU37/2011, was affected by zanamivir and RDE similarly to that of A/Udorn/72.

### Effect of Zanamivir on the Neutralization Activity of Saliva in the Presence or Absence of Bacterial Neuraminidase

**Figure 7:**
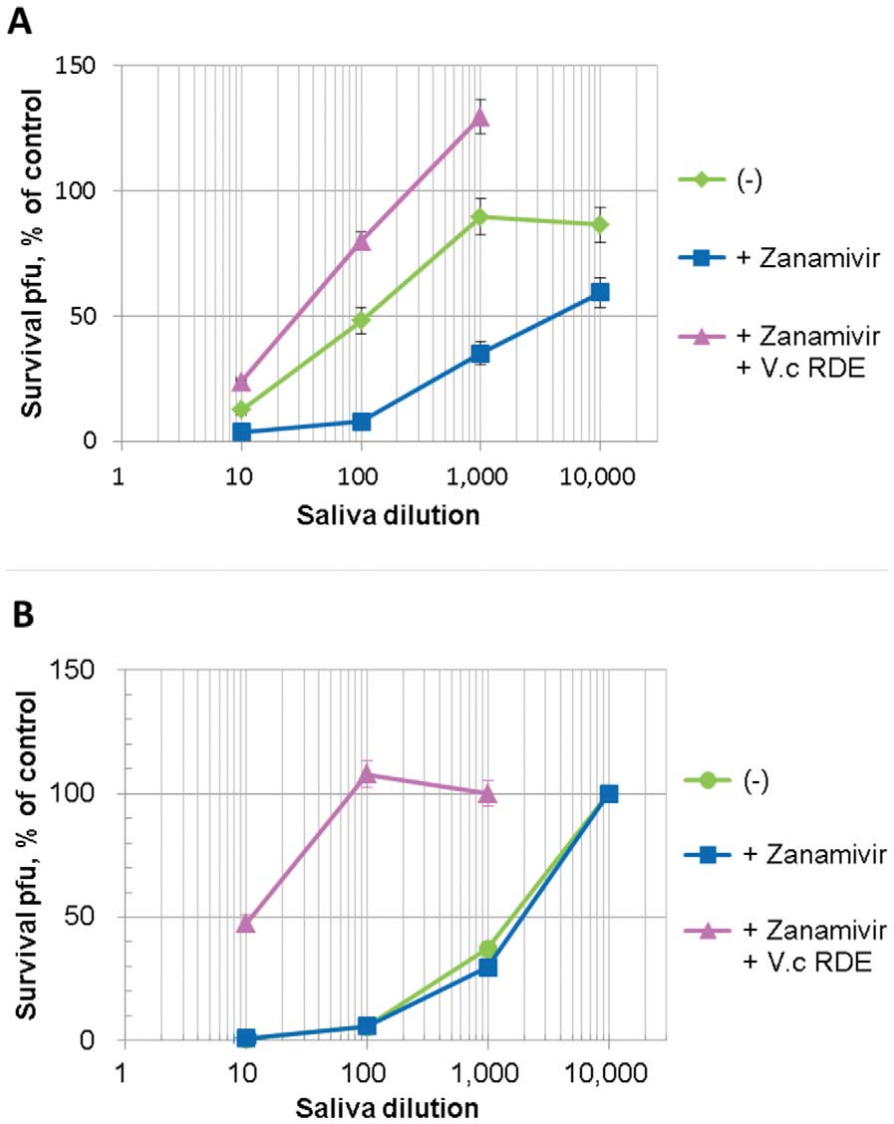
Effect of zanamivir and bacterial neuraminidase on the neutralization activity of saliva against influenza virus. Ten-fold serial dilutions of a saliva sample was mixed with an equal volume of A/Udorn/72 (**A**) and B/Johonnesburg/99 (**B**) virus suspensions (20,000 pfu/ml) containing zanamivir (+ Zanamivir, 500 nM) with or without *Vibrio cholerae* RDE (+ *V.c* RDE, 460 µunits/ml neuraminidase activity) and incubated at 37°C for 60 min. Survival infectivity (pfu) was determined by plaque assay.

As reported previously, we detected HI activity in saliva against various influenza viruses. Although this activity was correlated with the inhibition of infectivity [6], [31], quantitative titration of neutralization activity of saliva has not been reported yet. Therefore, we tested a saliva sample from one healthy donor for its neutralization activity against influenza A/Udorn//72 virus (**Figure 7A**). The saliva sample exhibited a neutralization titer of 1∶100 (the dilution which achieved 50% inhibition). The neutralization activity was affected by both zanamivir and bacterial neuraminidase similarly to the HI activity. The presence of 250 nM zanamivir enhanced the neutralization activity to 1∶4,000, while the presence of *V. cholerae* RDE (460 µunits/ml neuraminidase activity) attenuated the neutralization titer to 1∶30. Thus, the infectivity-neutralizing substance in saliva is sensitive to both NA of A/Udorn/72 and *V. cholerae* RDE. Interestingly, the enhancing effect of zanamivir on neutralization was marginal for the B/Johannesburg/99 virus (**Figure 7B**), where salivary neutralization activity was more than 10-fold higher against B/Johannesburg/99 (neutralization titer, 1∶1,500) than A/Udorn72. RDE decreased the neutralization titer by 150 fold to less than 1∶10, suggesting that the salivary neutralizing substance was resistant to neuraminidase of B/Johannesburg/99 virus but sensitive to *V. cholerae* RDE like the HA inhibitors. The similar responses of HI and neutralization activities of saliva to zanamivir and neuraminidases indicated that neutralization activity was dependent on HA inhibitors.

## Discussion

Neuraminidases are distributed in a variety of living organisms: influenza and parainfluenza viruses, bacteria, and animals. In the bacteria domain, *Diplococcus pneumoniae* [32], some groups of Streptococci [33], [34], *Vibrio cholerae* [35], *Corynebacteriumn diphtheriae* [36], and *Clostridiumn perfringens* [37] are known to produce neuraminidase. Rather recently, many bacteria isolated from human oral cavities or upper respiratory tracts, for instance, *Streptococcus mitis* [25], [29], *Streptococcus pneumoniae* [26], *Actinomyces naeslundii* and *Actinomyces viscosus* [27], *Porphyromonas gingivalis* [28], and *Streptococcus oralis* [30] were reported to secrete neuraminidases. We detected neuraminidase activity in the culture supernatants of nine strains of these 6 species (**Figure 1**). *S. pneumoniae* IID553 exhibited the highest activity among these. The activity of the culture supernatant was roughly the same level as that in the culture fluid of influenza A/Udorn/72 virus infected MDCK cells. Saliva samples also possessed neuraminidase activity. We hypothesized that the salivary neuraminidase activity would be derived from bacteria in the oral flora since secretions obtained directly from parotid and submandibular/sublingual duct orifices, the main sources of saliva, did not exhibit significant levels of the activity (unpublished data). However, we could not exclude the possibility of salivary neuraminidase originating from minor salivary glands.

Zanamivir inhibited the activity of influenza A and B viruses with IC_50_ values in the nanomolar range, which is consistent with previously reported values [38], and inhibited bacterial neuraminidases at concentrations above 10 µM (**Figure 2A**). The highly specific inhibition by zanamivir of influenza virus neuraminidase enabled us to assess the effect of bacterial neuraminidase on influenza virus infection. Inhibition of influenza virus NA results in attenuated virus release from infected cells [4], [39] and here we also confirmed that Zanamivir suppressed the yield of progeny virus in culture fluid of influenza virus-infected cells (**Figures 3** and **4**). The virus yield was completely restored by the addition of *S. pneumoniae* culture supernatant possessing neuraminidase activity. Nonspecific neuraminidase inhibitor DANA (2.5 mM) also suppressed virus yields but this suppression was not restored by *S. pneumoniae* culture supernatant since both viral and bacterial neuraminidases were inhibited. Highly active neuraminidases from *V. cholerae* (RDE) and *A. ureafaciens* also restored virus yields from the suppression by zanamivir (**Figure 4C**). These results clearly indicated that neuraminidase activity was responsible for the recovery of virus growth in the presence of the influenza virus NA-inhibitor drug.

Human saliva has been reported to contain hemagglutination inhibitors [5]–[7], [31]. In line with these reports, we detected high inhibitory activity in human saliva against hemagglutination activity and, in addition, infectivity of influenza A and B viruses (**Table 2**, **Figure 6** and **Figure 7**). The salivary infectivity-neutralization activity was enhanced in the presence of zanamivir for A/Udorn/72(H3N2), and *V. cholerae* RDE diminished the enhancement (**Figure 7A**). These results indicate that the viral NA plays a role in destroying soluble HA inhibitors in secretions and that bacterial neuraminidase could complement this destruction when viral NA is inhibited during drug treatment.

In summary, our results indicate that bacterial neuraminidases can functionally substitute for viral NA in terms of destroying virus receptors on both infected-cell surfaces and soluble hemagglutination inhibitors in salivary secretions. These findings imply that the effectiveness of NA inhibitor drugs, recently developed and commonly prescribed for influenza worldwide, may be antagonized by neuraminidases derived from bacteria flora in patients. In the clinical setting, the concentration of zanamivir in sputum 6 h after oral inhalation of 10 mg of zanamivir powder was 1,441 ng/ml, or 4.3 µM at most [40], while its concentration minutes after inhalation was calculated to be 5,870 ng/ml, or 17.5 µM at most. In other words, these concentrations are one to two log orders lower than the IC_50_ concentrations for bacterial neuraminidases (**Figure 2A**), indicating that the prescribed dose of zanamivir is not sufficient to inhibit bacterial neuraminidases. Therefore, if a certain amount of neuraminidase activity, originating from bacteria, is present on the surface of the respiratory tract, influenza virus infection, release and spread may not be suppressed by NA inhibitor drugs. In agreement with this possibility, it has been reported that receiving professional oral care and oral health guidance from a dental hygienist reduces both the number of oral bacteria and the activities of neuraminidase in saliva, resulting in a reduction in the risk of infection from influenza [41]. Altogether, the control of bacterial neuraminidases in the upper respiratory tract should be taken in consideration when using prescribed NA inhibitors in order to minimize reduced drug potency.

## Materials and Methods

### Ethics Statement

Protocols for experiments with human materials were approved by the Ethics Committee of Nihon University School of Medicine (Tokyo, Japan) (certification number 21-23-1, May 18, 2010). Participants provided their written informed consent to participate in this study.

### Reagents

Zanamivir (an influenza virus NA-inhibitor drug) was kindly provided by GlaxoSmithKline, Hertfordshire, UK. 2-Deoxy-2,3-dehydro-N-acetylneuraminic acid (DANA, a neuraminidase inhibitor reagent) was purchased from Sigma-Aldrich Co., St. Louis, MO, USA. Highly purified neuraminidase [EC 3.2.1.18] from *Arthrobacter ureafaciens* was purchased from Nacalai Tesque, Inc. Kyoto, Japan. Receptor-destroying enzyme, RDE (II), produced from *Vibrio cholerae* was purchased from Denka Seiken Co., Ltd., Tokyo, Japan.

### Virus Preparations

Influenza A/Udorn/307/72 (H3N2), A/Kitakyushu/159/93 (H3N2), A/Panama/2007/99 (H3N2), A/Puerto Rico/8/34 (H1N1), A/Yamagata/120/86 (H1N1), A/New Caledonia/20/99 (H1N1), A/Chiba/1001/2009 (H1N1)pdm (kindly provided by H. Eguchi and T. Ogawa, Chiba Prefectural Institute of Public Health), B/Johannesburg/5/99 and B/Kyoto/KU37/2011 viruses were propagated in MDCK cells in minimum essential medium (MEM; GIBCO, Gland Island, NY, USA ) containing 2.5 µg/ml TPCK-trypsin (Worthington Biochemical Corporation, Lakewood, NJ, USA) and penicillin and streptomycin (100 units/ml each, GIBCO, Gland Island, NY, USA) antibiotics at 34°C in 5% CO_2_.

### Saliva Samples

Saliva samples were collected from three healthy volunteers, taking no medications, by simple expectoration into 50 ml tubes. Mucinous precipitates were removed by centrifugation at 10,000×g for 5 minutes and bacteria were excluded by filtration with a syringe filter of 0.45 µm Supor membrane (PALL, Port Washington, NY, USA). Freshly prepared saliva samples were used for neuraminidase assays. Saliva samples were heated at 56°C for 30 min to inactivate neuraminidase activities before hemagglutination inhibition and infectivity neutralization assays.

### Bacterial Culture Supernatant Preparation

Streptococcus salivarius (JCM5707, ATCC9159), Streptococcus oralis (ATCC35037, GTC276), Streptococcus pneumoniae (ATCC33400, GTC26, IID553), Streptococcus pyogenes (GTC262), Streptococcus gordonii (ATCC10558), Streptococcus anginosus (ATCC3339T), Streptococcus mitis (ATCC6249, ATCC903, GTC495), Streptococcus sanguinis (ATCC10556), *Actinomyces naeslundii* (JCM8349), *Actinomyces viscosus* (ATCC15987) and *Porphyromonas gingivalis* (ATCC33277) were obtained from Japan Collection of Microorganisms (RIKEN BioResource Center, Saitama, Japan; JCM strains), American Type Culture Collection (Manassas, VA, USA; ATCC strains), Pathogenic Microorganism Genetic Resource Stock Center (Gifu University School of Medicine, Gifu, Japan; GTC strains), and Institute for Infectious Disease (The Institute of Medical Science, The University of Tokyo, Tokyo, Japan; IID strains). *Streptococcus mutans* (KMM7, KMM8, MT8148R, 007-1, 35-1, EM3, KMM5, 75-1), *Streptococcus sobrinus* (KSM6, AHT(g), KSM10, KSM9, KSM8, KSM1, 11-4) and *Streptococcus salivarius* (HT9R, HHT) were from our collection of human oral bacteria. To prepare culture supernatants, these bacteria were incubated in Trypticase soy broth (Becton, Dickinson and Company, Franklin Lakes, NJ, USA) overnight at 35°C under aerobic conditions, and the culture supernatants were clarified by centrifugation for 15 min at 1,750×g and filtrated by a syringe filter with a 0.45 µm Supor membrane (PALL, Port Washington, NY, USA) to exclude bacteria.

### Hemagglutination Assay

Two-fold serial dilutions of virus samples were made in 50 µl of phosphate buffered saline (PBS; GIBCO, Gland Island, NY, USA) in 96-well U-bottom plates (BD, Franklin Lakes, NJ, USA). To each well, 50 µl of 0.5% (v/v) chicken red blood cells (Nippon Biotest Laboratories, Tokyo, Japan) in PBS was added. The plates were kept at 4°C for 1 h, after which, the hemagglutination patterns were read and hemagglutination (HA) titers were determined from the last dilutions showing complete hemagglutination. One HA unit (HAU) was defined as the quantity of virus contained in 1 ml of virus suspension of HA titer 1.

### Hemagglutination Inhibition Assay

Two-fold serial dilutions of saliva samples were made in 25 µl of PBS on 96-well U-bottom plates. Then 25 µl of indicator viruses (8 HAU/ml PBS) were added for each dilution, and the plates were incubated for 1 h at 37°C. To each well, 50 µl of 0.5% (v/v) chicken red blood cells in PBS were added. The plates were kept at 4°C for 1 h and then the hemagglutination pattern was read. The reciprocal of the highest dilution that completely inhibited hemagglutination was taken as the hemagglutination inhibition (HI) titer.

### Neuraminidase Assay

Neuraminidase activity was measured using a commercially available chemiluminescent kit, NA-Star (Applied Biosystems, Foster City, CA, USA), which includes a chemiluminescent substrate, 1,2-dioxetane derivative of sialic acid (sodium(2-chloro-5-(4-methoxyspiro{1,2-dioxetane-3,2′-(5-chloro)tricyclo [3.3.1.1^3,7^]decan}-4-yl-phenyl-5-acetamido-3,5-dideoxy-α-D-glycero-D-galacto-2-nonulopyranoside)onate), following the manufacturer’s protocol with minor modifications. A volume of 10 µl of sample dilutions and 40 µl of NA-star assay buffer were added to each well of a 96-well plate. Then 10 µl of 0.01 mM NA-Star substrate was added to each well and incubated at 34°C for 30 min. The luminescence signals for triplicate samples were counted for 1.0 second with a 2.0 second delay after the injection of 60 µl of NA-Star accelerator using a LB941 Multimode Reader TriStar equipped with automatic injectors for the accelerator (Berthold Technologies GmbH & Co. KG., Bad Wildbad, Germany). Unit activity of sample neuraminidase was calculated using a standard curve obtained by a characterized neuraminidase, a highly purified neuraminidase from *Arthrobacter ureafaciens* (Nacalai Tesque, Kyoto, Japan) of known unit activity, where one unit is defined as the activity to release 1 µmol of N-acetylneuraminic acid from substrate of NAN-Lactose per min. Neuraminidase activity was expressed as the arbitrary units of luminescence signals or as calculated units.

### Neuraminidase Inhibition Assay with Zanamivir

Samples were diluted with PBS to give approximate neuraminidase activity of 5,000 arbitrary units of luminescence signals and mixed with an equal volume of ten-fold serial dilutions of zanamivir (from 10 mM to 0.1 nM in PBS) or DANA (from 10 mM to 0.1 µM in PBS). The final concentrations in the mixtures ranged from 5 mM to 0.05 nM for zanamivir and 5 mM to 0.05 µM for DANA. The mixtures were incubated at 37°C for 30 min and assayed for neuraminidase activity. The IC_50_ value was read on the inactivation dose response curve.

### Plaque Assay

The infectivity of viruses was determined by plaque assay as described below. Ten-fold serial dilutions of virus samples were made in Hanks balanced salt solution (HBSS; GIBCO, Gland Island, NY, USA) and 0.1 ml of the dilutions was inoculated on MDCK cell monolayers in 6-well tissue culture plates (Corning, Lowell, MA, USA). After adsorption of the virus onto the cells for 30 min at room temperature, 1.6 ml of Leibovitz’s L15 medium (GIBCO, Gland Island, NY, USA) containing 0.6% SeaKem ME agarose (Lonza, Basel, Switzerland), 1.5% gelatin (Nacalai, Kyoto, Japan) and 2.5 µg/ml TPCK-trypsin was added to each well and allowed to solidify. The plates were incubated for 3 days at 34°C and the number of plaques was counted. Infectivity was expressed as plaque forming units (pfu) per ml.

### Virus Growth Inhibition Assay

The inhibition of virus growth by neuraminidase inhibitors in the presence or absence of bacterial neuraminidase was assayed by measuring the released progeny virus in the culture fluid of infected cells. MDCK cells seeded on 12-well tissue culture plates were inoculated with 50 µl of virus suspension at a MOI of 0.001 or 0.01. After the adsorption for 1 h on ice, 1 ml MEM supplemented with 2.5 µg/ml TPCK-trypsin containing neuraminidase inhibitor and/or bacterial neuraminidase was added to each well. At the indicated times of incubation at 37°C in 5% CO_2_, culture media were harvested and the virus titers, performed in triplicate wells, were determined by plaque assay.

### Infectivity Neutralization Assay

The infectivity-neutralization activity of saliva samples were assessed by measuring plaque reduction. Ten-fold serial dilutions of saliva samples were mixed with an equal volume of virus suspension (approximately 10,000 pfu/ml) containing 500 nM zanamivir with or without *Vibrio cholerae* RDE (460 µunits/ml neuraminidase activity) and incubated for 1 h at 37°C. The mixtures were then titrated for infectivity by plaque assay. To avoid effects of zanamivir and RDE on plaque formation, cells were washed with 1.6 ml of HBSS after virus adsorption in the plaque assay. The dilution to give 50% reduction of pfu was estimated using the plaque-reduction curve and taken as the infectivity neutralization titer.

### Immunofluorescence

MDCK cells grown on 13-mm diameter coverslips were inoculated with A/Udorn/72 virus at a MOI of 0.01 and incubated for 60 min on ice. The cells were then incubated for 4, 8, 12, and 16 h at 37°C in 5% CO_2_ in MEM containing 250 nM zanamivir and/or *Vibrio cholerae* RDE (20 µunits/ml neuraminidase activity). The cells were fixed with 4% paraformaldehyde for 10 min followed by cold methanol (-30°C) for 5 min at room temperature. The fixed cells were incubated for 30 min with rabbit polyclonal antibody against purified A/Udorn/72 virions [42] in PBS containing 1% bovine albumin (fraction V, Reheis Chemical Company, Phoenix, Arizona, USA). After two washes, the cells were incubated for 30 min with Alexa Fluor 647 goat anti-rabbit IgG (Life Technologies Japan, Tokyo, Japan) in PBS containing 1% bovine albumin and Hoechst 33342 (to counterstain nuclei of all cells). The coverslips were mounted on a slide glass with ProLong Gold antifade reagent (Life Technologies Japan, Tokyo, Japan) and the cells were observed using a fluorescence microscope (BZ-8000, Keyence, Osaka, Japan). Acquired images were analyzed by using the software attached to BZ-8000.

## References

[1] Li W, Escarpe PA, Eisenberg EJ, Cundy KC, Sweet C, et al (1998) Identification of GS 4104 as an orally bioavailable prodrug of the influenza virus neuraminidase inhibitor GS 4071. Antimicrob Agents Chemother 42: 647–653. 9517946 10.1128/aac.42.3.647 PMC105512

[2] von Itzstein M, Wu WY, Kok GB, Pegg MS, Dyason JC, et al (1993) Rational design of potent sialidase-based inhibitors of influenza virus replication. Nature 363: 418–423. 8502295 10.1038/363418a0

[3] Palese P, Shaw ML (2007) Orthomixoviride: The virus replication. Knipe DM, Howley PM, editors. Philadelphia: Lippincotto Williams & Wilkins.

[4] Palese P, Compans RW (1976) Inhibition of influenza virus replication in tissue culture by 2-deoxy-2,3-dehydro-N-trifluoroacetylneuraminic acid (FANA): mechanism of action. J Gen Virol 33: 159–163. 978183 10.1099/0022-1317-33-1-159

[5] Boat TF, Davis J, Stern RC, Cheng PW (1978) Effect of blood group determinants on binding of human salivary mucous glycoproteins to influenza virus. Biochim Biophys Acta 540: 127–133. 638205 10.1016/0304-4165(78)90441-5

[6] White MR, Helmerhorst EJ, Ligtenberg A, Karpel M, Tecle T, et al (2009) Multiple components contribute to ability of saliva to inhibit influenza viruses. Oral Microbiol Immunol 24: 18–24. 19121065 10.1111/j.1399-302X.2008.00468.x PMC2848456

[7] Chen CH, Zhang XQ, Lo CW, Liu PF, Liu YT, et al (2010) The essentiality of alpha-2-macroglobulin in human salivary innate immunity against new H1N1 swine origin influenza A virus. Proteomics 10: 2396–2401. 20391540 10.1002/pmic.200900775 PMC2890046

[8] Matrosovich MN, Matrosovich TY, Gray T, Roberts NA, Klenk HD (2004) Neuraminidase is important for the initiation of influenza virus infection in human airway epithelium. J Virol 78: 12665–12667. 15507653 10.1128/JVI.78.22.12665-12667.2004 PMC525087

[9] Shope RE (1931) Swine influenza : III. Filtration experiments and etiology. J Exp Med 54: 373–385. 19869924 10.1084/jem.54.3.373 PMC2132000

[10] Taubenberger JK, Reid AH, Krafft AE, Bijwaard KE, Fanning TG (1997) Initial genetic characterization of the 1918 "Spanish" influenza virus. Science 275: 1793–1796. 9065404 10.1126/science.275.5307.1793

[11] Reid AH, Fanning TG, Hultin JV, Taubenberger JK (1999) Origin and evolution of the 1918 "Spanish" influenza virus hemagglutinin gene. Proc Natl Acad Sci U S A 96: 1651–1656. 9990079 10.1073/pnas.96.4.1651 PMC15547

[12] Morens DM, Taubenberger JK, Fauci AS (2008) Predominant role of bacterial pneumonia as a cause of death in pandemic influenza: implications for pandemic influenza preparedness. J Infect Dis 198: 962–970. 18710327 10.1086/591708 PMC2599911

[13] Louria DB, Blumenfeld HL, Ellis JT, Kilbourne ED, Rogers DE (1959) Studies on influenza in the pandemic of 1957–1958. II. Pulmonary complications of influenza. J Clin Invest 38: 213–265. 13620784 10.1172/JCI103791 PMC444127

[14] Louie JK, Acosta M, Winter K, Jean C, Gavali S, et al (2009) Factors associated with death or hospitalization due to pandemic 2009 influenza A(H1N1) infection in California. JAMA 302: 1896–1902. 19887665 10.1001/jama.2009.1583

[15] Dhanoa A, Fang NC, Hassan SS, Kaniappan P, Rajasekaram G (2011) Epidemiology and clinical characteristics of hospitalized patients with pandemic influenza A (H1N1) 2009 infections: the effects of bacterial coinfection. Virol J 8: 501. 22050645 10.1186/1743-422X-8-501 PMC3217982

[16] Tashiro M, Ciborowski P, Klenk HD, Pulverer G, Rott R (1987) Role of Staphylococcus protease in the development of influenza pneumonia. Nature 325: 536–537. 3543690 10.1038/325536a0

[17] Tashiro M, Ciborowski P, Reinacher M, Pulverer G, Klenk HD, et al (1987) Synergistic role of staphylococcal proteases in the induction of influenza virus pathogenicity. Virology 157: 421–430. 3029981 10.1016/0042-6822(87)90284-4

[18] McCullers JA, Bartmess KC (2003) Role of neuraminidase in lethal synergism between influenza virus and Streptococcus pneumoniae. J Infect Dis 187: 1000–1009. 12660947 10.1086/368163

[19] Peltola VT, McCullers JA (2004) Respiratory viruses predisposing to bacterial infections: role of neuraminidase. Pediatr Infect Dis J 23: S87–97. 14730275 10.1097/01.inf.0000108197.81270.35

[20] Gottschalk A (1957) Neuraminidase: the specific enzyme of influenza virus and Vibrio cholerae. Biochim Biophys Acta 23: 645–646. 13426178 10.1016/0006-3002(57)90389-x

[21] Burnet FM, Stone JD (1947) The receptor-destroying enzyme of V. cholerae. Aust J Exp Biol Med Sci 25: 227–233. 20270643 10.1038/icb.1947.33

[22] Liu C, Air GM (1993) Selection and characterization of a neuraminidase-minus mutant of influenza virus and its rescue by cloned neuraminidase genes. Virology 194: 403–407. 8480427 10.1006/viro.1993.1276

[23] Hughes MT, Matrosovich M, Rodgers ME, McGregor M, Kawaoka Y (2000) Influenza A viruses lacking sialidase activity can undergo multiple cycles of replication in cell culture, eggs, or mice. J Virol 74: 5206–5212. 10799596 10.1128/jvi.74.11.5206-5212.2000 PMC110874

[24] Thonard JC, Hefflin CM, Steinberg AI (1965) Neuraminidase activity in mixed culture supernatant fluids of human oral bacteria. J Bacteriol 89: 924–925. 14273690 10.1128/jb.89.3.924-925.1965 PMC277566

[25] Nonaka H, Ishikawa Y, Otsuka M, Toda K, Sato M, et al (1983) Purification and some properties of neuraminidase isolated from the culture medium of oral bacterium Streptococcus mitis ATCC 9811. J Dent Res 62: 792–797. 6575018 10.1177/00220345830620070301

[26] Scanlon KL, Diven WF, Glew RH (1989) Purification and properties of Streptococcus pneumoniae neuraminidase. Enzyme 41: 143–150. 2542012 10.1159/000469069

[27] Moncla BJ, Braham P (1989) Detection of sialidase (neuraminidase) activity in Actinomyces species by using 2'-(4-methylumbelliferyl)alpha-D-N-acetylneuraminic acid in a filter paper spot test. J Clin Microbiol 27: 182–184. 2643620 10.1128/jcm.27.1.182-184.1989 PMC267258

[28] Moncla BJ, Braham P, Hillier SL (1990) Sialidase (neuraminidase) activity among gram-negative anaerobic and capnophilic bacteria. J Clin Microbiol 28: 422–425. 2108991 10.1128/jcm.28.3.422-425.1990 PMC269635

[29] Beighton D, Whiley RA (1990) Sialidase activity of the "Streptococcus milleri group" and other viridans group streptococci. J Clin Microbiol 28: 1431–1433. 2199505 10.1128/jcm.28.6.1431-1433.1990 PMC267946

[30] Homer KA, Kelley S, Hawkes J, Beighton D, Grootveld MC (1996) Metabolism of glycoprotein-derived sialic acid and N-acetylglucosamine by Streptococcus oralis. Microbiology 142 (Pt 5): 1221–1230. 10.1099/13500872-142-5-1221 8704962

[31] Hartshorn KL, White MR, Mogues T, Ligtenberg T, Crouch E, et al (2003) Lung and salivary scavenger receptor glycoprotein-340 contribute to the host defense against influenza A viruses. Am J Physiol Lung Cell Mol Physiol 285: L1066–1076. 12871854 10.1152/ajplung.00057.2003

[32] Chu CM (1948) Enzymic action of viruses and bacterial products on human red cells. Nature 161: 607. 10.1038/161606b0 18916833

[33] Stewart FS, Steele TW, Martin WT (1959) The mechanisms involved in the production of red cell panagglutinability by streptococcal cultures. Immunology 2: 285–294. 13834585 PMC1423960

[34] Hayano S, Tanaka A (1969) Sialidase-like enzymes produced by group A, B, C, G, and L streptococci and by Streptococcus sanguis. J Bacteriol 97: 1328–1333. 5776530 10.1128/jb.97.3.1328-1333.1969 PMC249851

[35] Ada GL, French EL, Lind PE (1961) Purification and properties of neuraminidase from Vibrio cholerae. J Gen Microbiol 24: 409–425. 13681288 10.1099/00221287-24-3-409

[36] Warren L, Spearing CW (1963) Sialidase (Neuraminidase) Corynebacterium diphtheriae. J Bacteriol 86: 950–955. 14080806 10.1128/jb.86.5.950-955.1963 PMC278551

[37] Cassidy JT, Jourdian GW, Roseman S (1965) The sialic acids. VI. Purification and properties of sialidase from Clostridium perfringens. J Biol Chem 240: 3501–3506. 4284295

[38] Bantia S, Parker CD, Ananth SL, Horn LL, Andries K, et al (2001) Comparison of the anti-influenza virus activity of RWJ-270201 with those of oseltamivir and zanamivir. Antimicrob Agents Chemother 45: 1162–1167. 11257030 10.1128/AAC.45.4.1162-1167.2001 PMC90439

[39] Su CY, Wang SY, Shie JJ, Jeng KS, Temperton NJ, et al (2008) In vitro evaluation of neuraminidase inhibitors using the neuraminidase-dependent release assay of hemagglutinin-pseudotyped viruses. Antiviral Res 79: 199–205. 18453004 10.1016/j.antiviral.2008.03.002

[40] Peng AW, Milleri S, Stein DS (2000) Direct measurement of the anti-influenza agent zanamivir in the respiratory tract following inhalation. Antimicrob Agents Chemother 44: 1974–1976. 10858364 10.1128/aac.44.7.1974-1976.2000 PMC89995

[41] Abe S, Ishihara K, Adachi M, Sasaki H, Tanaka K, et al (2006) Professional oral care reduces influenza infection in elderly. Arch Gerontol Geriatr 43: 157–164. 16325937 10.1016/j.archger.2005.10.004

[42] Shimizu K, Mukaigawa J, Oguro M, Ono Y, Nakajima K, et al (1985) Inhibition of transcriptase activity of influenza A virus in vitro by anti-haemagglutinin antibodies. Vaccine 3: 207–210. 4060849 10.1016/0264-410x(85)90107-0

